# Sequence Analysis of the Second Internal Transcribed Spacer (ITS2) Region of rDNA for Species Identification of *Trichostrongylus* Nematodes Isolated From Domestic Livestock in Iran

**Published:** 2012

**Authors:** R Ghasemikhah, M Sharbatkhori, I Mobedi, EB Kia, M Fasihi Harandi, H Mirhendi

**Affiliations:** 1 Vali-e-asr Hospital, Arak University of Medical Sciences, Arak, Iran; 2 Department of Medical Parasitology & Mycology, School of Public Health; National Institute of Health Research, Tehran University of Medical Sciences, Tehran, Iran; 3 Laboratory Science Research Center, Golestan University of Medical Sciences, Gorgan, Iran; 4 Infectious Diseases Research Center, Department of Medical Parasitology and Mycology, School of Medicine, Golestan University of Medical Sciences, Gorgan, Iran; 5 Department of Parasitology, School of Medicine, Kerman University of Medical Sciences, Kerman, Iran

**Keywords:** *Trichostrongylus*, ITS2, Iran, Livestock

## Abstract

**Background:**

Infectivity of herbivores with *Trichostrongylus* nematodes is widespread in many countries, having a major economic impact on breeding, survivability, and productivity of domestic livestock. This study was carried out on *Trichostrongylus* species isolated from domestic livestock in order to develop an easy-to-perform method for species identification.

**Methods:**

*Trichostrongylus* isolates were collected from sheep, goat, cattle, and buffaloes in Khuzestan Province, southwest Iran. Primary species identification was carried out based on morphological characterization of male worms. PCR amplification of ITS2-rDNA region was performed on genomic DNA and the products were sequenced. Phylogenetic analysis of the nucleotide sequence data was conducted employing Bayesian Inference approach. Consequently, a restriction fragment length polymorphism (RFLP) profile was designed to differentiate *Trichostrongylus* species.

**Results:**

A consensus sequence of 238 nucleotides was deposited in the GenBank for Iranian isolates of *Trichostrongylus* species including *T. colubriformis*, *T. capricola*, *T. probolurus* and *T. vitrinus*. The designated RFLP using restriction enzyme *Tas*I could readily differentiate among species having different ITS2 sequence. The molecular analysis was in concordance with morphological findings.

**Conclusion:**

Phylogenetic analysis indicated a close relationship among the sequences obtained in this study and reference sequence of relevant species. ITS2-RFLP with *Tas*I is recommended for molecular differentiation of common *Trichostrongylus* species.

## Introduction

Infection of digestive tract of herbivores with *Trichostrongylus* nematodes are distributed throughout the world and are of major veterinary and economic importance because of their high prevalence, pathological outcomes of infection and impacts on animal products. Usually, herbivores can be infected by several species of *Trichostrongylus* nematodes, each with a different pathological consequence on the animal (1, 2). Therefore, precise differentiation of *Trichostrongylus* species is important for operative control programs of the parasite. Although conventional morphological methods for identification of *Trichostrongylus* spp. are quite reliable in *Trichostrongylus* males, they are laborious and cannot be used for recognition of females and eggs (3, 4). Until date, many studies have reported DNA-based methods as an effective approach to differentiate *Trichostrongylus* species (4–7). In this case, rDNA-ITS2 has been indicated as a useful and reliable region for species identification within *Trichostrongylus* species (6–8).

Iran had long been considered as a major focus of human and animal trichostrongylid infection in the world (9–11). Different species had been prevalent in ruminants throughout the country (9, 12, 13), among those the occurrence of seven species in human is documented (9) with highest prevalence in nomadic tribes (11). Therefore, molecular studies of *Trichostrongylus* species will be a useful approach to have a perspective on trichostrongylid nematodes of the country compared with those from other parts of the world.

The aim of this study was to compare sequences of ITS2 region of ribosomal DNA in *Trichostrongylus* species isolated from some domestic livestock in Iran with those published from reference *Trichostrongylus* sequences of other countries, and to establish an easy and reliable method to distinguish *Trichostrongylus* species by a PCR-restriction fragment length polymorphism (RFLP) approach.

## Materials and Methods

### Parasite

Worms were collected from abomasums and small intestines of sheep, goats, cattle and buffalos from different abattoirs of Khuzestan Province, southwest of Iran. The samples were washed extensively in distilled water and preserved in ethanol 70%. The species of male nematodes were identified based on morphological characterizations, using a light microscope and nematodes taxonomic identification keys (14, 15).

### DNA extraction and Polymerase Chain Reaction

DNA was obtained from individual male *Trichostrongylus* nematodes. An isolate refers to a single male worm was obtained from any different hosts. Briefly, each individual single worm was crushed between two microscopic slides for 1 min with 300µl lysis buffer (NaCl 0.1M, EDTA 0.01M, Tris-HCl 0.1M, Triton X-100 2%, SDS 2%). The lysate from each sample was treated by 30 µg/ml proteinase K in 56 °C for an hour. Then, the DNA was extracted by conventional phenol/chloroform extraction and ethanol precipitation (16). Finally, the DNA pellet was eluted in 20 µl deionized distilled water and inserted at -20 °C until more analysis.

ITS2 fragment of ribosomal DNA was PCR-amplified, using forward (NC1: 5-ACGTCTGGTTCAGGGTTGTT-3) and reverse (NC2: 5-TTAGTTTCTTTTCCTCCGCT-3) primer pair (17). The PCR reactions were performed in a 25 µl volume containing, 12.5 µl of 2× premix (Ampliqon, Skovlunde, Denmark), 25 pmol of each primer, 10.5 DDW and 1 µl of extracted DNA in a thermocycler (Corbett Research, Sydney, Australia). The PCR program was one cycle of 95 °C for 6 min followed by 35 cycles of 94 °C for 45 seconds, 60 °C for 90 seconds and 72 °C for 60 seconds with a final extension of 72 °C for 5 min. Samples with 1µl DDW instead of templates were used in each run as negative controls.

Two microliters of each amplification product was conducted to 1.5% agarose gel electrophoresis in TBE buffer (90 mM Tris, 90 mM boric acid and 2 mM EDTA) at 100 V for 1 h. The gels included 0.5 µg/ml ethidium bromide (Roche, Mannheim, Germany) for staining. A 100-bp DNA ladder (Fermentas, Vilnius, Lithuania) was run to estimate the size of DNA in gels. The bands were visualized and photographed under UV light employing a transilluminator (UVItec, UK).

### Sequencing and phylogenetic analysis

Three or four PCR products of each *Trichostrongylus* species from different livestock hosts were subjected to sequencing, using above-mentioned primers. The results were analyzed by ClustalX (18) and DNASIS (Hitachi, Tokyo, Japan) softwares and were compared with relevant sequences related to sequences deposited in GenBank, using BLAST (http://www.ncbi.nlm.nih.gov/). The levels of sequence difference (D), were obtained, using pairwise comparison of a consensus representative sequence containing 238 nucleotides from different *Trichostrongylus* species found in the current study, via the formula of D=1-(M/L) (19), in which M is the number of alignment positions at which the two sequences had a common base, and L is the total number of alignment positions over which the two sequences are compared. For better understanding of relationship among isolates from Iran and other countries, all the different *Trichostrongylus* sequences described here conducted to a phylogenetic reconstruction along with reference sequences of *Trichostrongylus* species deposited in the GenBank from previous studies (6, 8, 20, 21), and an ITS2 sequence of *T. colubriformis* in the GenBank from Iran (AN: HQ389232). The analysis performed by Bayesian Inference approach employing MrBayes software v.3.1.2 (http://mrbayes.csit.fsu.edu/index.php). *Trichostrongylus tenuis*, the prevalent species in birds, was used as the outgroup. The tree was run several times to obtain the most frequent topology.

### RFLP

The sequences were subjected to *in-silico* cutting with almost all known restriction enzymes using DNASIS software. Consequently, *Tas*I has been considered to differentiate *Trichostrongylus* species. Five microliters of PCR product, 0.5 µl (5 units) of *Tas*I enzyme (Fermentas, Vilnius, Lithuania), 1.5 µl of 10X supplied buffer and 8 µl double distilled water, were incubated at 65 °C for 3 h. Restriction fragments were separated on 2.2% agarose gel in TBE buffer, stained with ethidium bromide and photographed****.

## Results

Among 107 individual male worms of *Trichostrongylus* collected in the present study, four species were identified morphologically. *Trichostrongylus colubriformis* was the most identified species. All extracted DNA samples successfully yielded an expected 320 bp PCR product. No PCR amplification was seen in negative controls.

**Fig. 1:**
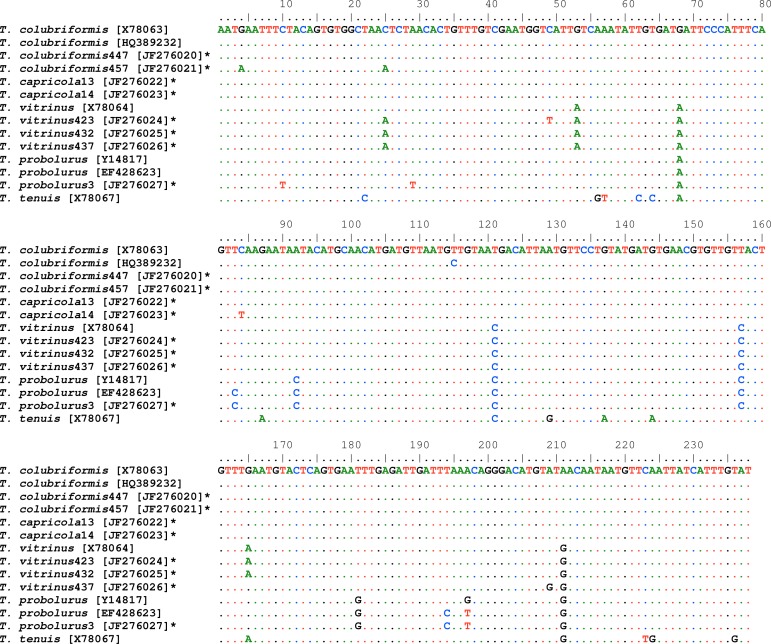
Alignments of the ITS2 sequences representing for all *Trichostrongylus* isolates in the present study with key reference sequences for *Trichostrongylus* species from previous studies (6, 8, 20–22). The accession numbers of individual sequences are given in square parentheses. Sequences with the bold asterisk inferred from this study. *T. tenuis* was used as the outgroup

**Fig. 2:**
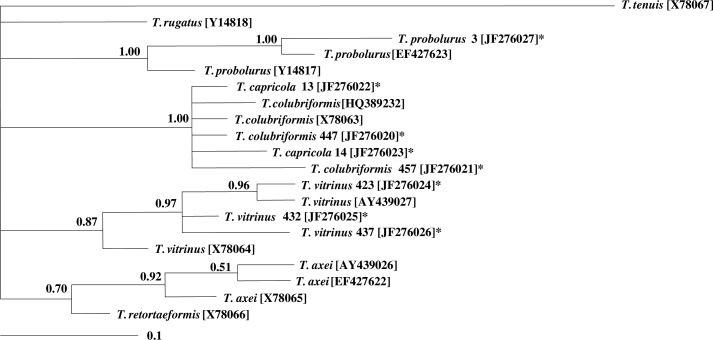
Genetic relationships of *Trichostrongylus* isolates from Iran and reference sequences for different species of *Trichostrongylus* selected from previous studies (6, 8, 20, 21). The titles with asterisk inferred from the present study. The relationships were inferred based on phylogenetic analysis of ITS2 sequence data using Bayesian Inference. Nodal support is given as a pp value. The scale bar indicates distance.

After sequencing, eight ITS2-sequences inferred from *Trichostrongylus* isolates, including two *T. colubriformis*, two *T. capricola*, three *T. vitrinus* and one *T. probolurus*. A consensus sequence containing 238 nucleotides for each individual sequence was deposited in GenBank (accession numbers: **JF276020** to **JF276027**). Figure 1 shows multiple alignment of the sequences. Based on pairwise comparison, the differences among all of the different sequences of ITS2 ranged 0.5–3.4% (data not shown). The consensus phylogenetic tree indicated four major groups with high statistical supports (PP: 0.76 to 1.00). All *Trichostrongylus* species obtained in this study were in a cluster with the relevant reference sequences from previous studies (Fig. 2).

The expected fragments after digesting the ITS2-PCR products of different species, by *Tas*I restriction enzyme, are showed in Table 1.

**Table 1:** The obtained fragments after *in silico* cutting of ITS2 region in common *Trichostrongylus* species. Relevant GenBank accession numbers was included

Species	Fragments size after digestion by *Tas*I	GenBank accession numbers
***T. colubriformis***	24, 45, 85, 174	**JF276020, JF276021**
***T. capricola***	24, 45, 85, 174	**JF276022, JF276023**
***T. vitrinus***	24, 45, 63, 85, 111	**JF276024, JF276025, JF276026**
***T. probolurus***	24, 63, 85, 156	**JF276027**

**Fig. 3:**
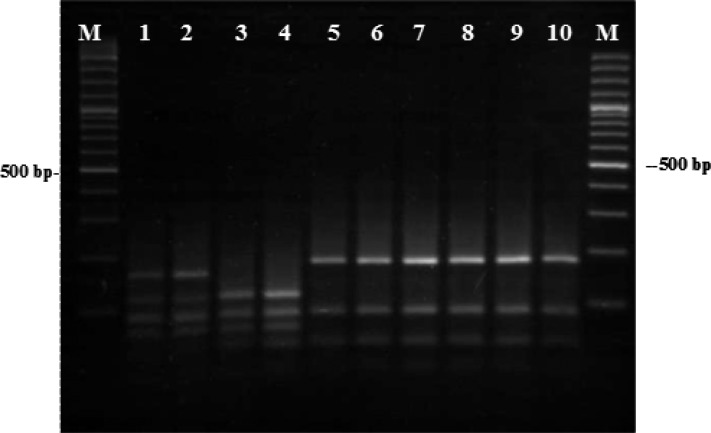
The pattern of PCR products after digestion with *TasI*: lanes 1–2 are *T. probolurus*, lanes 3–4 are *T. vitrinus*, lanes 5-7 and 8-10 are *T. capricola* and *T. colubriformis*, respectively. Lanes M, 100-bp DNA ladder

After RFLP analysis, the restriction enzyme *Tas*I produced three different patterns as expected based on *in-silico* analysis; one relevant to both *T. colubriformis* and *T. capricola*, one to *T. vitrinus* and another to *T. probolurus* (Fig. 3). In RFLP-gels, detected *Trichostrongylus* species were in concordance with morphology and sequences results. Not any intra-specific variation was seen in the RFLP patterns of the species with multiple isolates.

## Discussion

Molecular characterization of ITS2-rDNA region in four *Trichostongylus* species including *T. colubriformis*, *T. capricola*, *T. probolurus* and *T. vitrinus* collected from southwest Iran, were studied. All these species have been previously reported both in human (9–11, 23–24) and domestic livestock (9) in Iran. Furthermore, a recent study indicated that *T. colubriformis* was the main zoonotic species causing helminth infection in a rural village in Laos (24). In the present study, these species were studied using sequence analysis of ITS2-rDNA region and the sequence data for *T. capricola*, *T. vitrinus and T. probolurus* are submitted to the GenBank for the first time from Iran. Recently, a complete ITS1-5.8S-ITS2 rDNA sequence of *T. colubriformis* was deposited in the GenBank from Iran (**HQ389232**). This sequence has one nucleotide difference in ITS2 sequence with reference sequence of *T. colubriformis* (**X78063**) and also one of *T. colubriformis* ITS2 sequences from this study [**JF276020**]. The mentioned sequence was placed in one group along with other *T. colubriformis* and *T. capricola* sequences, having maximal statistical support (pp: 1.00) in the phylogenetic tree. Surprisingly, the ITS2-rDNA sequences of *T. capricola* isolates indicated 100% (**JF276022**) and 99.6% (**JF276023**) homology with reference sequence for *T. colubriformis* (**X78063**). Probably it can be explained with high similarity of these two species in morphological characters. As there is not any previous data for ITS2-rDNA sequence of *T. capricola* in the the GenBank to compare with the findings of the present study, the phylogenetic tree yields an overview of relationship of this species with other species. Two *T. capricola* sequences (**JF276022** and **JF276023**) were placed in one cluster along with two *T. colubriformis* sequences from the present study (**JF2760220** and **JF276021**), as well as the reference sequences for *T. colubriformis* (**X78063**), having maximum statistical support (pp= 1.00). The three *T. vitrinus* sequences (**JF2760224**- **JF276026**) indicated more relationship to *T. vitrinus* from UK (**AY439027**) (pp= 0.89) rather than those from Australia (**X78064**) (pp= 0.88). In addition, the sequence of *T. probolurus* was in a cluster with *T. probolurus* from Russia (**EF427623**), and these two were placed in an outer cluster with *T. probolurus* from Australia (**Y14817**), both clusters supported by maximum pp (1.00).

The PCR-RFLP approach has been used previously to distinguish among species of trichostrongylid nematodes, including *T. colubriformis*, *T. axei*, *T. vitrinus*, *Haemonchus contortus*, *Teladorsagia circumsinata* and *Cooperia oncophora* (7). RFLP analysis in current study could differentiate the *Trichostrongylus* species with different sequences. Due to high sequence similarity of *T. colubriformis* and *T. capricola*, these two species produced same RFLP pattern.

The present study represents preliminary sequence information of *Trichostrongylus* species from Iran. Few numbers of sequenced *Trichostrongylus* isolates and using one DNA locus (ITS2) to characterize the isolates were limitations in the current study. The *T. capricola* isolates could not be distinguished from *T. colubriformis* by ITS2-RFLP indicating the sequence is identical to *T. colubriformis*, whereas alignments of *T. colubriformis* sequences recently added to the GenBank (**AB503241**-**52**) from a village in Laos, with two *T. capricola* sequences (**AF210006** and **AF210030**) originally from France, demonstrates that these two species have clear differences in 28s rDNA sequences (24, 25). As, trichostrongylid species are distributed all throughout the country, in different livestock (8), and by means of traditional methods the occurrence of seven species in human have been reported (9), utilization of molecular tools employing sequences of multiple genes will benefit understanding of the rate of zoonocity for each species, as well as obtaining a better perspective of *Trichostongylus* species in various host species in different geographical areas.
